# Performance Analysis of Hospital Managers Using Fuzzy AHP and Fuzzy TOPSIS: Iranian Experience

**DOI:** 10.5539/gjhs.v8n2p137

**Published:** 2015-06-11

**Authors:** Milad Shafii, Seyed Mostafa Hosseini, Mohammad Arab, Ezzatollah Asgharizadeh, Fereshteh Farzianpour

**Affiliations:** 1 Department of Health Management and Economics, School of Public Health, Tehran University of Medical Sciences, Tehran, Iran; 2 Department of Epidemiology and Biostatistics, Tehran University of Medical Sciences, Tehran, Iran; 3 Department of Industrial Management, Faculty of Management, University of Tehran, Tehran, Iran

**Keywords:** FAHP, FTOPSIS, hospital managers, performance

## Abstract

**Background and Objectives:**

Hospitals are complex organizations that require strong and effective management. The success of such organizations depends on the performance of managers. This study provides a comprehensive set of indicators to assess the performance of hospital managers in Iranian Ministry of Health owned hospitals.

**Methods:**

This research was a cross-sectional study. First, reviewing the literature and using experts’ viewpoints and convening a panel of experts, the dimensions of performance have been selected and came in the form of a performance model. Then, using Fuzzy Analytic Hierarchy Process (FAHP), the chosen dimensions were weighted. Finally, based on the weighted performance dimensions, a questionnaire was designed and after confirming the reliability and validity, through a census, 407 senior and middle managers from 10 hospitals in Yazd, Iran completed it and performance of CEO_s_ in these hospitals was evaluated using the Fuzzy Technique for Order Preference by Similarity Ideal Solution (FTOPSIS).

**Results:**

To measure the performance of hospital managers, a performance assessment model consisted of 19 sub-dimensions in 5 main dimensions (Functional, Professional, Organizational, Individual and Human) was developed. The functional area had the most weight and the individual area had the least weight, as well. The hospital managers had different performance levels in each category and sub-dimensions. In terms of overall performance, the hospital managers C and H had the best and the worst performance, respectively.

**Conclusions:**

The use of appropriate dimensions for performance, prioritizing them and evaluating the performance of hospital managers using appropriate techniques, can play an effective role in the selection of qualified managers, identifying strengths and weaknesses in performance and continuous improvement of them.

## 1. Introduction

The success of any organization to achieve its goals is highly dependent on the performance of the organization managers (Gharaei, 2004). managers play an important role in resource allocation, improving the quality of services and ultimately promoting the organizational performance (Stefl, 2008). The issue becomes more notable at the case of health care organizations dealing with human lives (Akbari, Tofighi, Torabi, & Arab, 2005). In fact, the role of hospital managers is different from the role of managers of other organizations and industries. They both require similar skills and knowledge in management and organization development (Shewchuk, O’Connor, & Fine, 2005). On the other hand, given that the highest share of health budget is allocated to health organizations especially hospitals and these organizations are important levers of health system for delivering health services, and managers of these organizations are responsible for operating the goals and visions determined by policy-makers in order to enhance the welfare and well-being of the community, therefore, the appropriate evaluation of managers plays a crucial role in the proper use of the costs and health promotion (Nikoukar, Ketabi, & Moazam, 2010; Pillay, 2010). While the existence of an effective and efficient evaluation system is crucial for identifying talents and potential capabilities of managers, but unfortunately, no formal and systematic evaluation system has been applied to evaluate the performance of hospital managers using rapid, informal and nonobjective assessment tools, would lead assessors to perform subjective evaluation (Shahkordi, 2003; Dadgar, Janati, Tabrizi, Asghari-Jafarabadi, & Barati, 2012). If the performance evaluation system of hospital managers become efficient and tangible containing measurable indicators for assessment, many of the costs can be reduced in hospitals and better services can be delivered to the population. That’s why provision of good services depends on good and effective management (Aozora Bank, 2002; Calhoun et al., 2008). However, one of the most important steps in the evaluation process is definition of accurate and feasible criteria (Fazli & Azar, 2002). Therefore, in order to improve the performance of hospital managers, there is a need to evaluate them based on a sound conceptual model. Such model facilitates the selection and training of managers by defining an appropriate set of skills and competencies which in turn may contribute to efficiency, effectiveness, and responsiveness in the health care organizations (Pillay, 2010; Liang, Leggat, Howard, & Koh, 2013). In the majority of international studies which recently have been conducted, competency-based models have been used to assess the competency and performance of healthcare managers (Pillay, 2010; Calhoun et al., 2008; Khadka, Gurung, & Chaulagain, 2013; Wongprasit, 2014; Anderson & Pulich, 2002; Lockhart & Backman, 2009; American College of Healthcare Executives [ACHE], 2015). Several studies have been conducted to assess the competency and performance and provide dimensions of competency and performance appropriate to the activities of managers in the health field in different countries (Dadgar et al., 2012; Shewchuk et al., 2005; M^ac^kinnon et al., 2005; Landry, Stowe, & Haefner, 2012). Determining appropriate and prioritized dimensions and indicators to evaluate the performance of hospital managers in an appropriate model and taking into account local conditions, is an important affair that this study seeks to answer it. To evaluate the performance of hospital CEO_s_, the FAHP technique has been applied for determining the weights of performance dimensions and the FTOPSIS has been used to rank the hospital managers. These techniques have been employed in many studies to evaluate the performance and have been emphasized on the effectiveness of them (Avazpour, Ebrahimi, & Fathi, 2013; Sun, 2010; Islam & Rasad, 2006; Patil & Kant, 2014; Tsai, Chang, & Lin, 2010; AKKOÇ & Vatansever, 2013; Fang, Chang, & Chen, 2010). Considering all the above, this article aims to propose a model for evaluating the performance of hospital managers and analyzed the performance of ten hospital administrators in Yazd, Iran using FAHP and FTOPSIS techniques.

## 2. Methods

### 2.1 Evaluation Framework

The study was conducted in analytical cross sectional form during the period of March 2014 to February 2015 in province of Yazd, Iran and analyzed the performance of hospital managers. We used the qualitative-quantitative approach in this research. In qualitative approach, we reviewed the literature, got feedback from the experts by email and hold the expert panel. In quantitative approach, we used Fuzzy AHP and Fuzzy TOPSIS techniques to analyze the data. First, with review of the research literature in the fields of competency and performance evaluation and with a focus on hospital administrators, a list of performance dimensions were obtained. In order to achieve a conceptual framework for performance evaluation, a questionnaire containing performance dimensions and indicators derived from the literature review was designed, and was sent to 20 experts of the health care management and performance evaluation (including managers of hospitals and health care networks, faculty members of healthcare management departments and experts involved in the evaluation of performance at the university level, and the Ministry of Health).

**Figure 1:**
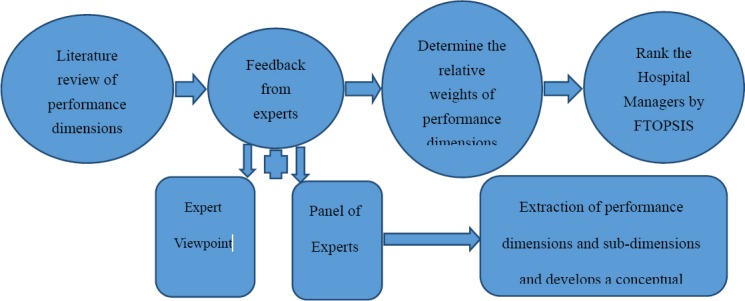
Overall framework of research

After collecting the viewpoints of the experts in relation to the proposed aspects and dimensions, holding a 7-member panel of experts (including three administrators of the hospital, two faculty members of healthcare management department and two experts of performance evaluation), the final dimensions and sub-dimensions of performance were classified and the necessary consensus was obtained on the conceptual framework of performance for hospital managers, so the final model and its application to evaluation of hospital managements’ performance was supported by additional literature review, experts viewpoints and expert panel inputs thus ensuring content validity (Pillay, 2008). Then the paired comparisons questionnaire in order to prioritizing and weighing areas and subareas of the final performance model were designed, and in order to be completed, were presented to 21 of stakeholders who somehow are affected or related to the performance of the managers. Then using FAHP technique, the relative weight of each dimension and sub-dimension was obtained. In the next step, an 83-item questionnaire was developed from the conceptual model and after confirming the reliability (Cronbach’s Alpha = 0.96) and validity (the questionnaire was evaluated by eight experts with an interest in hospital management to identify potential problems related to misunderstanding or misinterpretation of questions to ensure face validity), the CEO_s_ performance of 10 hospitals in province of Yazd, Iran, using the FTOPSIS technique and by all senior executives of hospitals including the head of the hospital, the hospital administrator, director of nursing services and all middle managers (Total of 407) were compared together and managers were ranked in terms of performance. Figure 1 shows the overall framework of research

### 2.2 Fuzzy Set Theory

Fuzzy set theory, was introduced by Zadeh (1965) to deal with vague problems and incorporating imprecise data into the decision framework. If x is an issue, Ã is a subset of X such that for all x∈X is. Here μ_Ã_ (x)∈[0,1] is the numbers that have been allocated to membership x to Ã and μ_Ã_(x)is called the membership Ã function. In this study we used triangular fuzzy number Ã that can be defined by a triplet (a, b, c) as illustrated in Figure

**Figure 2:**
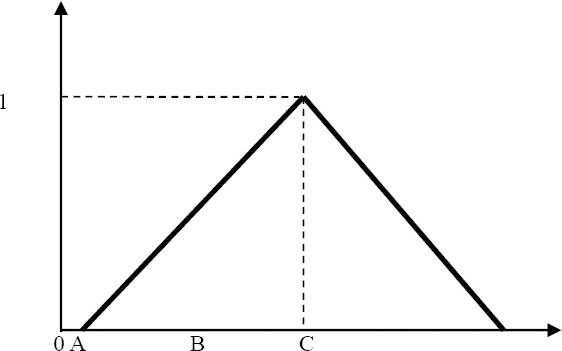
A triangular fuzzy number Ã

Membership functions defined as follows:


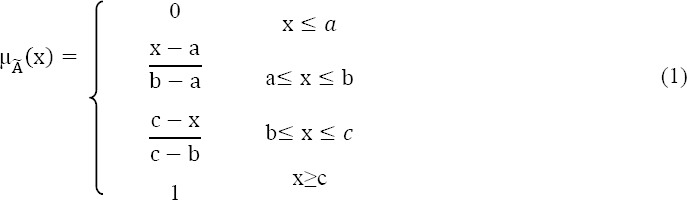


To calculate the total distance between two fuzzy numbers and we used the following equation:


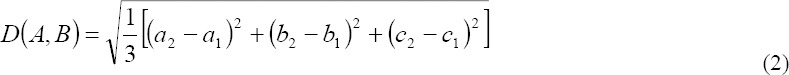


If the two numbers, where a_1_ ≤ b_1_ ≤ c_1_ and, where a_2_ ≤ b_2_ ≤ c_2_ are considered as fuzzy numbers, then the addition and subtraction the two numbers shown as follows:


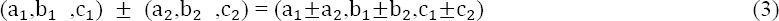


And to multiply and divide the two numbers of the equation we use:


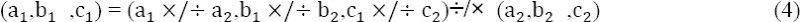


The merit of using triangular fuzzy numbers is their intuitive and computational-efficient representation and appropriateness to quantify the vague and imprecise information in most decision problems including human resource selection (e.g. rating for creativity, personality, leadership) (Karsak, 2002; Avazpour et al., 2013).

### 2.3 Fuzzy Analytic Hierarchy Process

Analytical hierarchy process (AHP) is a powerful decision-making methodology for calculating the weights and priority of different criteria. This technique first proposed by Saaty (1980). AHP technique is often criticized, because of its inability to manage the inherent uncertainty and imprecision in the pairwise comparison process (Deng, 1999). FAHP was proposed by Van Laarhoven and Pedrycz (1983) to overcome these shortcomings because it’s more accurate to give interval judgments than fix value judgments (Avazpour et al., 2013).The procedure of FAHP summarized as follows:

Step 1: construct the hierarchy of decision problem

Step 2: Definition of fuzzy numbers for doing paired comparisons.

Step 3: Construct the Paired comparison matrix using fuzzy numbers.


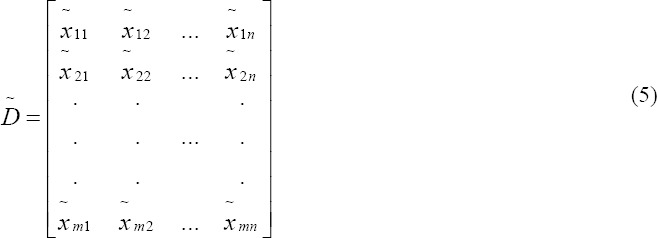


Step 4: Calculate *S_i_* for each row of the paired comparison matrix


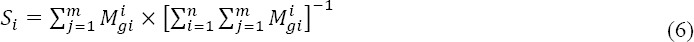


In above equation, i is the number of row and j is the number of column.


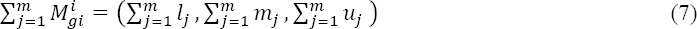



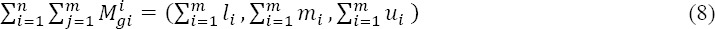



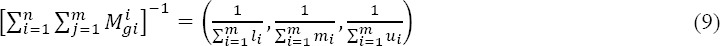


In the relations, *l_i_,m_i_ and u_i_* are respectively the first to third components of fuzzy numbers.

Step 5: Calculate the large degree of *S_i_* relative to each other

If *M_1_=(l_1_,m_1_, u_1_)* and *M_2_=(l_2_,m_2_, u_2_)* are two triangular fuzzy numbers:


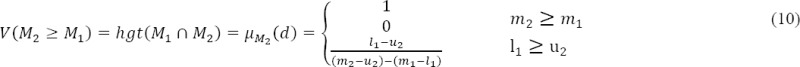


Step 6: Calculate the weight of criteria and options in the paired comparison matrix


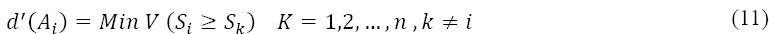


Step7: Calculate the final weight


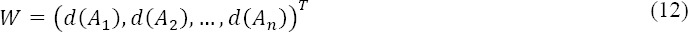


### 2.4 FTOPSIS methodology

The TOPSIS method is based on the principle that the selected alternative should have the shortest distance from the positive ideal solution and the longest distance from the negative ideal solution. FTOPSIS is the extension of TOPSIS to the fuzzy environment (Kuo, Tseng, & Huang, 2007; Yang & Hung, 2007).The mathematical concepts and FTOPSIS procedure borrowed from (Torfi et al., 2010; Sun, 2010; Avazpour et al., 2013) and summarized as follows:

Step 1: Construct the fuzzy performance/decision matrix and choose the appropriate linguistic variables for alternatives with respect to criteria:


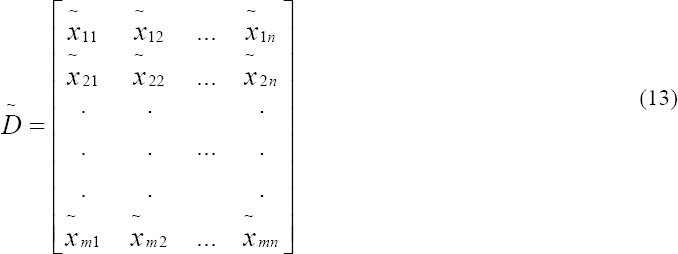



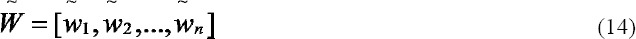


X̃*_ij_*: The performance rating of the ith alternative with respect to the jth criterion which has been calculated as following:


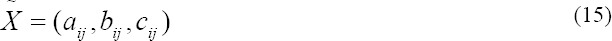


W̃*_ij_*: The rate of individual’s viewpoint’s importance which is expressed as following:


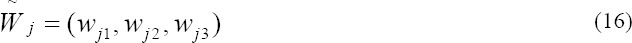


It should be noted that in this study, because of the same level of experts viewpoints’ importance, for the whole target population was defined as fallowing:


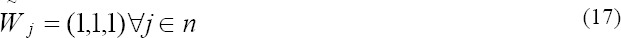


Step 2: Normalize the fuzzy decision matrix:

In this step the fuzzy decision matrix of individuals’ viewpoints should be converted to a fuzzy normalized matrix. To obtain this matrix the fallowing function can be used:


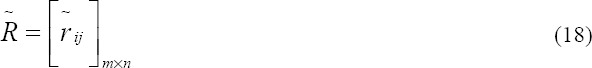



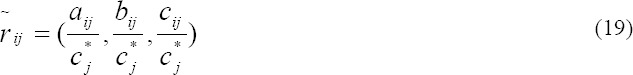


In this function (relationship) C_j_ for each individual is equal to:


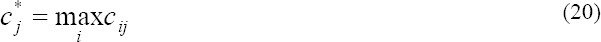


Step3: Calculate the weighted normalized fuzzy decision matrix:


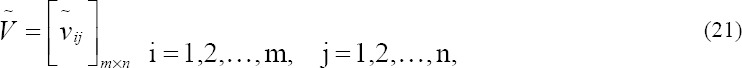



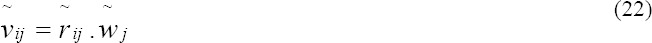


Step 4: determine the fuzzy positive- ideal solution and fuzzy negative- ideal solution for the alternatives.


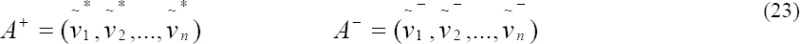


We used the fuzzy positive ideal solution and the fuzzy negative ideal solution as:


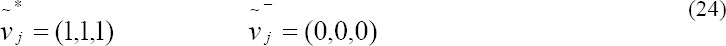


Step 5: Calculate the sum distances of each alternative from FPIS and FNIS: If Aand B are two fuzzy number as follows, then the distance between these fuzzy numbers obtained by the following equation:


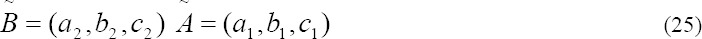



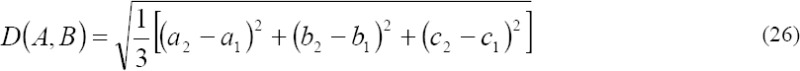


Given the above description about calculating method of distance between two fuzzy numbers, the distance of each alternative from positive and negative ideals can be calculated as below:


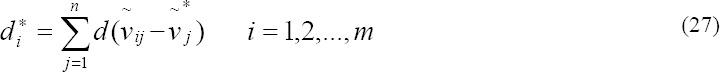



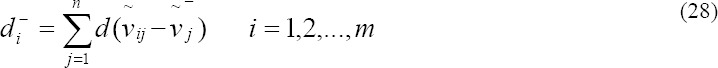


Step 6: Calculate the relative proximity of the ith alternative from the positive ideal. Relative proximity is defined as:


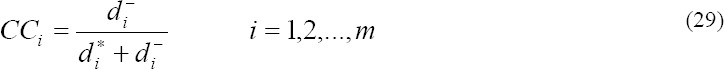


Step 7: options (choices) ranking: Options of an assumed problem can be ranked according to the descending order of Ci.

## 3. Results

### 3.1 The Conceptual Framework for Evaluating the Performance of Hospital Administrators

To extract performance dimensions for hospital administrators, first, a comprehensive review was done through searching in various databases. Then using feedback from the experts, the final conceptual framework containing the performance areas and subareas were determined (Table 1).

**Table 1:** Main dimensions and sub-dimensions of hospital administrators’ performance

Main dimensions	sub-dimensions
**Functional:** using a set of skills, abilities, means, procedures and techniques necessary to perform the main duties of a manager	**Planning**: a process including a set of tasks to achieve certain objectives and includes goal setting, finding how to achieve it, design of optimal status in future and finding ways and means to achieve it.
	**Organizing**: a process in which through the division of labor among individuals and work groups and facilitating coordination between them, is strived to achieve the organizational purposes and implementing things in an effective and efficient way.
	**Leadership**: creating motivation, inner desire and inspiration for subordinates in order to move towards the vision and common organizational goals
	**Control**: a process in which the current performance is continually measured in order to ensure that the predetermined objectives are fulfilled and enables managers to detect deviations and take corrective action to deal with them.
**Professional (professionalism):** attempt to act on the opinions, values, ethics and individual and organizational principles and also trying to continually improve and further education	**individual Professional behavior**: efforts to act on the basis of individual beliefs, values, ethics and principles
	**organizational Professional behavior**: try to act on the basis of organizational beliefs, values, ethics and principles
	**Training and continuous improvement at the Individual level**: efforts to continuous improvement and individual education in order to develop and expand the capabilities of the manager in terms of his/her career.
	**development and continuous improvement at the organizational level:** efforts toward continuous improvement and development of the organization in its various service fields
**Human:** ability to establish effective interpersonal relationships which are essential to the working environment, creating communication networks, transmission and receipt of information or ideas clearly and effectively, and also try to understand concerns, feelings and thoughts of employees, patients and the society and respecting them and understanding their needs to safety as a human	**Patient-orientation (commitment to patient):** Given the high respect and importance to the patient, his/her needs and demands
	**Communication Management**: Using different techniques and approaches to establish and maintain effective communication in oral and written forms with different people and also the creation of social and communication networks with effective individuals and groups in order to enhance cooperation and their support of programs and organizational goals
	**Human safety**: respecting the individual as a human and understand the importance of ensuring their safety as well as having the ability and readiness to guarantee the safety of patients, staff and other community members who are linked in some way to the hospital.
**Organizational:** The ability to correctly and optimally use of organizational resources including information resources, human resources, physical and financial resources	**Information and Information Technology management**: determining informational needs, collecting, organizing, processing and providing appropriate, updated, timely and reliable information using new methods and technologies
	**Human Resources Management**: knowledge of the status of available human resources and decision making about how to Recruitment, maintain, and optimize the use of human resources in a safe, efficient and effective manner
	**Financial Resources Management**: knowledge of the status of available financial resources and decision making about how to capture, collect, allocate, maintain and optimize the use of financial resources in a safe, efficient and effective manner
	**Physical resource management**: Knowledge of the status of available physical resources including buildings, facilities and equipment and decision making about how to buy, build, maintain and optimize the use of physical resources in a safe, efficient and effective manner
**Individual**: Having positive personal characteristics both in terms of personality and complex mental abilities	**General characteristics**: a set of internal and personality characteristics that have a positive impact on carrying out better the roles and responsibilities
	**Creativity and innovation**: shaping the self-metal thoughts to create a new idea or concept and the application of that idea or concept into a plan, innovation or a new product or service
	**Analysis and comprehension**: the optimal use of high-level mental processes such as thinking, remembering, understanding the causality relationships and analysis in order to understand the phenomenon that a person is faced with
	**Decision making**: evaluation of different solutions to solve the problem and making the right decision at the right time in order to solve the problem

The performance aspects achieved from the literature review, expert viewpoints and panel of experts, were placed in the 5 categories and 19 sub-dimensions. Five main dimensions including functional (consists of four sub-dimensions of planning, organizing, leading and controlling), professional (including 4 sub-dimensions of individual professional behavior, organizational professional behavior, training and continuous improvement at the individual level and development and continuous improvement at the organizational level), human (including three sub-dimensions of patient-orientation, communication management and human safety); organizational (including 4 sub-dimensions of information management, human resources management, financial resources management and physical resources management) and individual (including 4 sub-dimensions of general characteristics, creativity and innovation, analysis and comprehension and decision-making), respectively.

### 3.2 Determining the Weight of Dimensions and Sub-Dimensions of Hospital Administrators’ Performance

The paired comparison questionnaire of the performance areas and subareas was provided to 21 experts and using the FAHP, the weight and rank of performance areas and subareas was obtained (Table 2).

**Table 2:** Weight and rank of main dimensions and sub-dimensions using FAHP

Main dimensions	Weight	Rank	Sub-dimensions	Weight	Rank
Functional	0.3735	1	Planning	0.2843	2
			Organizing	0.1438	4
			Leadership	0.3166	1
			Controlling	0.2553	3
Professional	0.1327	3	Individual professional behavior	0.1610	4
			Organizational professional behavior	0.2351	2
			Training and continuous improvement at the Individual level	0.1613	3
			development and continuous improvement at the organizational level	0.3426	1
Human	0.3123	2	Patient-orientation	0.3189	3
			Communication management	0.3477	1
			Human safety	0.3334	2
Organizational	0.1153	4	Information and information technology management	0.2105	3
			Human resources management	0.4293	1
			Financial resources management	0.2618	2
			Physical resources management	0.0984	4
Individual	0.0662	5	General characteristics	0.1977	3
			Creativity and innovation	0.1607	4
			Analysis and comprehension	0.2557	2
			Decision making	0.3859	1

As Table 2 shows, dimensions of functional (0.3735) and individual (0.0662) have the most and least weight, respectively. In addition, in the dimension of functional, the sub-dimension of leadership had the most weight (0.3166) and the sub-dimension of organizing had the least weight (0.1438). In the main area of professionalism, the development and continuous improvement at the organizational level was the first priority (with the weight of 0.3426) and the individual professional behavior was the last (with the weight of 0.1610). In the human category, the communication management (with the weight of 0.3477) and the patient-orientation (with the weight of 0.3189) have gained the most and least significance, respectively. In the organizational dimension, the human resources management (0.4293) and management of physical resources (0.0984) have gained the highest and lowest weight, respectively. In the individual area, the decision-making had the highest priority (with the weight of 0.3859) and the innovation and creativity had the lowest priority (with the weight of 0.1607), as well.

After determining the weight of performance dimensions, the performance assessment questionnaire was distributed between the respondents and performance of hospital managers was evaluated according to each of the dimensions and sub-dimensions.

### 3.3 Performance Evaluation and Ranking of Hospital Administrators According to Each of the Performance Sub-Dimensions Within the Main Dimensions

Table 3 shows the performance score and rank of hospital administrators in each of the functional sub-dimensions. The hospital administrator C has gained the highest rank in all four areas of planning, leadership, organizing and controlling, respectively. While the lowest performance score and rank in the area of planning is related to the hospital administrator I and in other three areas, the hospital administrator H has achieved the lowest rank

**Table 3:** Performance status of hospital managers in functional sub-dimensions

	performance score of hospital managers in functional sub-dimensions												Performance rank of HM_s_ in functional sub-dimensions			

	Planning			Leadership			Organizing			Controlling		
Hospital Managers											
	d_i_^+^	d_i_^-^	CC_i_	d_i_^+^	d_i_^-^	CC_i_	d_i_^+^	d_i_^-^	CC_i_	d_i_^+^	d_i_^-^	CC_i_	Planning	Leadership	Organizing	Controlling
HM-A	0.3437	0.8609	0.2853	0.5241	0.5643	0.4815	0.4894	0.5968	0.4505	0.5020	0.5825	0.4629	3	2	2	2
HM-B	0.1837	1.0987	0.1432	0.4094	0.6784	0.3763	0.3937	0.6902	0.3632	0.4027	0.6731	0.3743	7	7	7	6
HM-C	0.6496	0.5519	0.5406	0.6437	0.4441	0.5917	0.6246	0.4629	0.5744	0.6128	0.4638	0.5692	1	1	1	1
HM-D	0.2114	1.0361	0.1694	0.4260	0.6619	0.3916	0.3839	0.7046	0.3526	0.3722	0.7066	0.3450	5	6	8	9
HM-E	0.1690	1.1144	0.1317	0.3853	0.7004	0.3549	0.4186	0.6647	0.3864	0.4053	0.6723	0.3761	8	9	6	5
HM-F	0.2086	1.0786	0.1621	0.4513	0.6394	0.4137	0.4626	0.6281	0.4241	0.3865	0.6931	0.3580	6	5	4	8
HM-G	0.3939	0.8069	0.3281	0.4678	0.6215	0.4294	0.4376	0.6549	0.4005	0.4391	0.6461	0.4046	2	3	5	4
HM-H	0.1576	1.1683	0.1189	0.2911	0.8026	0.2661	0.3136	0.7857	0.2853	0.3106	0.7868	0.2830	9	10	10	10
HM-I	0.1409	1.1968	0.1053	0.3992	0.6869	0.3676	0.3809	0.7124	0.3484	0.3945	0.6885	0.3643	10	8	9	7
HM-J	0.2846	0.9309	0.2341	0.4628	0.6228	0.4263	0.4747	0.6104	0.4374	0.4805	0.5965	0.4461	4	4	3	3

The performance status of the studied administrators in the sub-dimensions of professionalism in Table 4 also shows that the hospital administrator C had the best performance in all four sub-dimensions of training and continuous improvement at the individual level, continuous improvement and development at the organizational level, organizational professional behavior and individual professional behavior.

**Table 4:** Performance status of hospital managers in professional sub-dimensions

	performance score of hospital managers in professional sub-dimensions												Performance rank of HM_s_ in professional sub-dimensions			

	Individual Professional B			Organizational Professional B			Development and Improvement(Org)			Training and Improvement (Ind)		
Hospital Managers																
	d_i_^+^	d_i_^-^	CC_i_	d_i_^+^	d_i_^-^	CC_i_	d_i_^+^	d_i_^-^	CC_i_	d_i_^+^	d_i_^-^	CC_i_	T&I (Ind)	D&I (org)	OPB	IPB
HM-A	0.5020	0.5825	0.4629	0.4338	0.6601	0.3966	0.4784	0.6048	0.4417	0.3994	0.6830	0.3690	5	3	2	2
HM-B	0.4027	0.6731	0.3743	0.3465	0.7372	0.3198	0.3841	0.6891	0.3579	0.3814	0.6926	0.3551	6	6	6	6
HM-C	0.6128	0.4637	0.5692	0.6391	0.4440	0.5900	0.6096	0.4650	0.5672	0.6012	0.4735	0.5594	1	1	1	1
HM-D	0.3722	0.7066	0.3450	0.3430	0.7429	0.3158	0.4227	0.6570	0.3915	0.4136	0.6516	0.3883	3	4	7	9
HM-E	0.4053	0.6723	0.3761	0.3195	0.7792	0.2908	0.2714	0.8161	0.2495	0.2797	0.8091	0.2569	9	9	9	5
HM-F	0.3865	0.6931	0.3580	0.3049	0.7926	0.2778	0.3746	0.6943	0.3504	0.3157	0.7611	0.2931	8	8	10	8
HM-G	0.4391	0.6461	0.4046	0.4191	0.6742	0.3833	0.4840	0.6022	0.4456	0.4790	0.6057	0.4416	2	2	3	4
HM-H	0.3106	0.7868	0.2830	0.3242	0.7725	0.2956	0.2664	0.8114	0.2471	0.2491	0.8291	0.2310	10	10	8	10
HM-I	0.3945	0.6885	0.3643	0.3569	0.7324	0.3277	0.3815	0.7015	0.3522	0.3306	0.7460	0.3071	7	7	5	7
HM-J	0.4805	0.5965	0.4461	0.3967	0.6950	0.3633	0.3986	0.6835	0.3683	0.4068	0.6749	0.3761	4	5	4	3

While the lowest performance in the three sub-areas, including Individual training and continuous improvement, organizational continuous improvement and development and individual professional behavior, belongs to the hospital administrator H and in the field of organizational professional behavior, this has belonged to the hospital administrator F.

Table 5 shows the performance status of the hospital managers in human sub-dimensions. Based on the results in Table 5, the hospital administrator C has gained the highest score and rank in the three sub-areas of human safety, patient-orientation and communication management and the hospital administrator H has gained the lowest score and rank in all three human subareas.

**Table 5:** Performance status of hospital managers in human sub-dimensions

Hospital Managers	performance score of hospital managers in human sub-dimensions									Performance rank of HM_s_ in human sub-dimensions		

	Communication management			Patient-Orientation			Human safety					

	d_i_^+^	d_i_^-^	CC_i_	d_i_^+^	d_i_^-^	CC_i_	d_i_^+^	d_i_^-^	CC_i_	Human safety	Patient-Orientation	Communication management
HM-A	0.5053	0.5776	0.4666	0.5146	0.5661	0.4761	0.4577	0.6248	0.4228	2	2	4
HM-B	0.4420	0.6377	0.4093	0.3833	0.6893	0.3573	0.3719	0.7041	0.3457	6	6	7
HM-C	0.6232	0.4556	0.5776	0.6438	0.4390	0.5945	0.6229	0.4611	0.5746	1	1	1
HM-D	0.5345	0.5381	0.4983	0.4339	0.6420	0.4033	0.3966	0.6792	0.3686	3	4	2
HM-E	0.3526	0.7316	0.3252	0.3039	0.7837	0.2794	0.3324	0.7577	0.3049	9	9	9
HM-F	0.5158	0.5690	0.4754	0.3537	0.7289	0.3267	0.3333	0.7501	0.3076	8	8	3
HM-G	0.5025	0.5801	0.4641	0.4930	0.5906	0.4549	0.3888	0.7073	0.3547	5	3	5
HM-H	0.2781	0.8105	0.2555	0.2418	0.8383	0.2238	0.2833	0.8075	0.2597	10	10	10
HM-I	0.3797	0.7086	0.3489	0.3759	0.7029	0.3484	0.3527	0.7328	0.3249	7	7	8
HM-J	0.4955	0.5854	0.4584	0.4028	0.6812	0.3715	0.3998	0.6879	0.3675	4	5	6

Also, as the performance of the hospital managers in organizational subareas is shown in Table 6, the hospital administrator C has gained the highest score and rank in all four subareas of human resources management, information management, physical resources management and financial resources management.

**Table 6:** Performance status of hospital managers in organizational sub-dimensions

	performance score of hospital managers in organizational sub-dimensions												Performance rank of HM_s_ in organizational sub-dimensions			

	Human Resource Management			Information Management			Physical Resource Management			Financial Resource Management						
Hospital Managers																
	d_i_^+^	d_i_^-^	CC_i_	d_i_^+^	d_i_^-^	CC_i_	d_i_^+^	d_i_^-^	CC_i_	d_i_^+^	d_i_^-^	CC_i_	HRM	IM	PRM	FRM
HM-A	0.4793	0.6134	0.4386	0.4613	0.6216	0.4259	0.5318	0.5511	0.4910	0.5379	0.5555	0.4919	2	3	2	2
HM-B	0.3619	0.7267	0.3325	0.3749	0.6987	0.3492	0.4005	0.6821	0.3700	0.4043	0.6824	0.3721	5	6	8	7
HM-C	0.6674	0.4249	0.6109	0.7079	0.3702	0.6565	0.6595	0.4248	0.6082	0.6096	0.4815	0.5587	1	1	1	1
HM-D	0.3961	0.695	0.3630	0.4157	0.6575	0.3873	0.4027	0.6766	0.3731	0.4015	0.6858	0.3692	4	4	7	8
HM-E	0.3217	0.7811	0.2917	0.2998	0.7860	0.2761	0.3377	0.7495	0.3106	0.3195	0.7818	0.2901	8	9	9	10
HM-F	0.3445	0.7547	0.3134	0.3171	0.7524	0.2965	0.4611	0.6266	0.4239	0.3942	0.7104	0.3568	6	8	3	9
HM-G	0.3079	0.7790	0.2833	0.4751	0.6070	0.4390	0.4298	0.6448	0.3999	0.4809	0.5936	0.4475	9	2	5	3
HM-H	0.3067	0.7921	0.2791	0.2303	0.8472	0.2137	0.2924	0.8003	0.2676	0.4172	0.6723	0.3829	10	10	10	5
HM-I	0.3404	0.7588	0.3097	0.3258	0.7541	0.3017	0.4119	0.6717	0.3801	0.4111	0.6798	0.3768	7	7	6	6
HM-J	0.4150	0.6790	0.3793	0.4100	0.6683	0.3802	0.4357	0.6461	0.4027	0.4593	0.6332	0.4204	3	5	4	4

The lowest score and rank of the performance in three sub-dimensions of human resources management, information management and physical resources management belonged to the hospital administrator H and in sub-dimension of financial resources management; it belonged to the hospital administrator E. The performance status of the hospital managers in individual sub-dimensions is shown in Table 7. It indicates that the first performance rank belonged to the hospital administrator C in all four sub-dimensions of comprehension, decision making, creativity and general characteristics. The lowest performance has been belonged to the hospital administrator H in each of the four sub-dimensions, as well.

**Table 7:** Performance status of hospital managers in individual sub-dimensions

	performance score of hospital managers in individual sub-dimensions												Performance rank of HM_s_ in individual sub-dimensions			

Hospital Managers	Analysis and comprehension			Decision making			Creativity and innovation			General characteristics		
	
	d_i_^+^	d_i_^-^	CC_i_	d_i_^+^	d_i_^-^	CC_i_	d_i_^+^	d_i_^-^	CC_i_	d_i_^+^	d_i_^-^	CC_i_	Analysis	Decision making	creativity	characteristics
HM-A	0.4530	0.6307	0.4180	0.4425	0.6414	0.4082	0.4348	0.6525	0.3998	0.4577	0.6249	0.4228	2	5	3	2
HM-B	0.3804	0.6942	0.3540	0.3906	0.6834	0.3637	0.3698	0.7080	0.3431	0.3719	0.7040	0.3456	5	7	5	6
HM-C	0.6190	0.4558	0.5759	0.7030	0.3748	0.6522	0.6407	0.4362	0.5949	0.6229	0.4611	0.5746	1	1	1	1
HM-D	0.3643	0.7070	0.3401	0.3905	0.6776	0.3656	0.3801	0.6996	0.3520	0.3965	0.6792	0.3686	6	6	4	3
HM-E	0.2880	0.7949	0.2660	0.3148	0.7753	0.2888	0.3316	0.7576	0.3044	0.3323	0.7577	0.3049	9	9	7	9
HM-F	0.3632	0.7309	0.3319	0.5024	0.5779	0.4650	0.3307	0.7411	0.3085	0.3333	0.7501	0.3076	7	2	6	8
HM-G	0.4188	0.6700	0.3846	0.4664	0.6186	0.4298	0.3271	0.7525	0.3030	0.3888	0.7073	0.3547	4	3	8	5
HM-H	0.2450	0.8364	0.2265	0.2346	0.8439	0.2175	0.2437	0.8377	0.2253	0.2833	0.8075	0.2597	10	10	10	10
HM-I	0.3543	0.7268	0.3277	0.3347	0.7473	0.3093	0.2967	0.7852	0.2742	0.3526	0.7328	0.3249	8	8	9	7
HM-J	0.4292	0.6491	0.3980	0.4467	0.6370	0.4122	0.6840	0.5459	0.5561	0.3997	0.6879	0.3675	3	4	2	4

### 3.4 Performance Evaluation and Ranking of Hospital Managers According to Each of the Main Performance Dimensions

Table 8 shows the performance status and rank of the studied hospital administrators according to each of the main performance areas, where the hospital administrator C has gained the highest score and rank in all the main areas (functional, professional, human organizational and individual) and the hospital administrator H has gained the lowest score and rank.

**Table 8:** Performance status of hospital managers in each of the main dimensions

Hospital Managers Main dimensions		HM-A	HM-B	HM-C	HM-D	HM-E	HM-F	HM-G	HM-H	HM-I	HM-J
Functional 0/3735	d_i_^+^	0.4577	0.3719	0.6229	0.3965	0.3323	0.3333	0.3888	0.2833	0.3526	0.3997
	d_i_^-^	0.6248	0.7040	0.4611	0.6792	0.7577	0.7501	0.7073	0.8075	0.7328	0.6879
	CC_i_	0.4228	0.3456	0.5746	0.3686	0.3049	0.3076	0.3547	0.2597	0.3249	0.3675
	Rank	2	6	1	3	9	8	5	10	7	4
Individual 0/0662	d_i_^+^	0.4454	0.3820	0.6623	0.3779	0.3104	0.3875	0.4147	0.2407	0.3307	0.4328
	d_i_^-^	0.6403	0.6945	0.4147	0.698	0.7649	0.6916	0.6685	0.8395	0.7515	0.6492
	CC_i_	0.4102	0.3548	0.6149	0.3512	0.2886	0.3590	0.3828	0.2228	0.3056	0.4000
	Rank	2	6	1	7	9	5	4	10	8	3
Organizational 0/1153	d_i_^+^	0.4694	0.3882	0.6594	0.4339	0.3225	0.3644	0.4496	0.2591	0.3468	0.4244
	d_i_^-^	0.6132	0.6873	0.4203	0.639	0.7642	0.7110	0.6363	0.8251	0.7367	0.6571
	CC_i_	0.4336	0.3609	0.6107	0.4044	0.2967	0.3388	0.4140	0.2389	0.3201	0.3924
	Rank	2	6	1	4	9	7	3	10	8	5
Professional 0/1327	d_i_^+^	0.492	0.3867	0.6207	0.3883	0.3834	0.4143	0.4622	0.3148	0.3676	0.4576
	d_i_^-^	0.5986	0.7014	0.4667	0.7006	0.7036	0.6782	0.6306	0.7856	0.7251	0.6292
	CC_i_	0.4511	0.3554	0.5707	0.3566	0.3527	0.3792	0.4229	0.2860	0.3364	0.4210
	Rank	2	7	1	6	8	5	3	10	9	4
Human 0/3123	d_i_^+^	0.3993	0.3814	0.6011	0.4135	0.2796	0.3156	0.4789	0.2491	0.3306	0.4068
	d_i_^-^	0.6829	0.6925	0.4735	0.6516	0.8091	0.7611	0.6056	0.8290	0.7460	0.6748
	CC_i_	0.3689	0.3551	0.5594	0.3882	0.2568	0.2931	0.4416	0.2310	0.3070	0.3760
	Rank	5	6	1	3	9	8	2	10	7	4

### 3.5 The Overall Performance Evaluation and Final Ranking of Hospital Managers

Table 9 shows the overall performance score and final ranking of the studied hospital administrators. According to the results, in terms of the overall performance, the hospital administrator C has gained the first performance rank within the ten hospital managers (with the performance score of 0.5860) and the hospital administrator H has achieved the last rank (with the performance score of 0.2577), as well.

**Table 9:** Overall performance status and final ranking of the hospital managers

Hospital managers Main dimensions		HM-A	HM-B	HM-C	HM-D	HM-E	HM-F	HM-G	HM-H	HM-I	HM-J
Overall Performance	d_i_^+^	0.4692	0.3843	0.6344	0.4042	0.3370	0.3814	0.4488	0.2810	0.3539	0.4351
	d_i_^-^	0.6174	0.6972	0.4481	0.6753	0.7503	0.7028	0.6391	0.8093	0.7332	0.6496
	CC_i_	0.4318	0.3553	0.5860	0.3744	0.3099	0.3517	0.4125	0.2577	0.3255	0.4011
	Rank	2	6	1	5	9	7	3	10	8	4

## 4. Discussion

In this study, we analyzed the performance status of hospital administrators using a set of qualitative and quantitative research methods. In the first step, through the comprehensive review of research literature and getting feedback from the experts, a set of performance indicators for hospital managers which were categorized in five main dimensions (functional, organizational, professional, individual and human) and 19 sub-dimensions were achieved. Then, using the technique of FAHP and expert viewpoints, the importance and weight of each major and minor dimension were determined. Functional and individual area achieved the most and least weight, respectively.

In a study to evaluate the employees’ performance, after determining the performance indicators of employees using FAHP, two indicators of personal characteristics and interpersonal relationships gained the most weight (Avazpour et al., 2013), while in present study, the main dimension of individual has gained the least weight in comparison to other major areas of performance, but, the human scope, usually considered as interpersonal competency in different studies, has been ranked the second in terms of importance and weight and it is relatively consistent with the intended study. The results of the study by Fang which calculated the weight of five main areas of competency for middle health managers using AHP, shows that two dimensions of personality and individual capabilities have achieved the highest and lowest rank respectively, that the results of which are largely inconsistent with the results of this study (Fang et al., 2010). However, the difference in weight of some common indicators in different studies was more because the different experts have different views and perspectives in relation to the importance of performance indicators and it is quite normal. After calculating the weight of indicators, the performance of the hospital managers was analyzed using FTOPSIS Technique and results presented according to each of the major and minor performance areas and overall performance of managers. The empirical results of a study show that the FAHP and FTOPSIS techniques are practical approaches to solve problems, especially when the performance criteria and rankings are vague and imprecise (Torfi et al., 2010).

The studied managers obtained different ranks in different areas of their performance. Managers of some hospitals had better performance according to the selected criteria, while some other managers’ status was different that in some cases, the performance difference was remarkable. However, other studies have been conducted on the performance evaluation of managers and definition of performance and competency dimensions.

Dadgar et al. (2012) suggested required competencies of managers in seven categories of planning, organizing and managing employees’ performance, leadership, information management, clinical governance, resource management and performance indicators which have many similarities with the sub-dimensions of the proposed model in this study (Dadgar et al., 2012). This is probably due to the similar environments of these two research and common social and cultural conditions. In addition, similarity in the competencies required by managers for better management of hospitals in Iran in both research, represents the high generalizability of the proposed model, especially for Iran.

A survey of 358 rural primary care managers in Southern Thailand, found six critical managerial competencies (Mohd-Shamsudin & Chuttipattana, 2012). These include visionary leadership, planning and evaluation, health promotion and disease prevention, information management, collaboration and ultimately communications that some of these competencies such as planning and evaluation, information management and communications are sub-dimensions of present study. Another study presented a model of leadership competencies for managers of private hospitals in Thailand (Wongprasit, 2014). In this study using the grounded theory approach, the snowball sampling and in-depth interviews with 30 managers of private hospitals in Thailand, 26 leadership competencies required by hospital managers, have been categorized into 6 dimensions of 1-individual 2-management 3-people 4- medicine 5- sense of balanced management and 6- direction. Research by Gue (2002) proposed three groups of interpersonal, informational and decision making roles and six vital and important roles including leadership, communication, monitoring, strategic entrepreneurship, control and allocation of resources; for senior healthcare managers (Guo, 2002).

Research by Chadwell (2012) which was conducted to assess the training needs of hospital managers in Nepal assessed the opinions of 103 managers with different expertise from 31 hospitals. This study proposed six factors such as strategic management, financial management, service management, people management, information management and self-management (Chadwell et al., 2012). Anderson and Pulich (2002) focused on the role of managerial traditional functions and tasks including the four main task of planning, organizing, leading and controlling to enable organizations achieving their goals and classified the managerial competencies into 4 main functional areas required for all managers and supervisors in order to fulfill their roles in modern health care organizations (Anderson & Pulich, 2002). Each of the conducted studies, considered different aspects of manager performance that some of them are included in main performance dimensions of this study and some others were among the sub-dimensions. Regarding to all items mentioned, it can be expected that the performance dimensions obtained in this study have a suitable comprehensiveness and are applicable, as well. In this study to collect data, ethical approval was obtained from the research ethics committee of Sadoughi University of medical sciences in Yazd. Also we gave participants information about how their data will be used and ensure confidentiality of their information and identity.

A strength point of this study comparing with similar studies, is having a comprehensive view, yet specialized toward the performance of hospital administrators and the integration of Fuzzy Logic and Multi-Criteria Decision Making Techniques in order to minimize the impact of the subjective nature of the issue on the main aim of the study.

However, this study also had limitations that among them are the need for attention to the type of hospital in terms of being specialized or general and its potential impact on the managers’ performance which this necessitates studies with more details and taking into account the effect of variable, the hospital type, in the results of evaluating the performance of hospital managers. Another important point about the limitations of this study refers to the subjective nature of evaluating the performance that although efforts have been made to greatly solve this problem through integrating the Fuzzy Logic with techniques used in this study, impact of this problem cannot be ignored. Although, we tried to choose the right time and place for distribution of questionnaires between respondents and allocate the enough response time to them, however, we can’t ensure the accuracy and focus of respondents in answering to questions.

At the practical level, considering the importance of giving feedback in performance assessment process, we recommend that assessors develop a formal evaluation framework for hospital managers and pay attention to the timeframe of feedback after evaluation to help the hospital managers for improving their skills and performance. Also, holding formal and short-term training courses based on the identified strengths and weaknesses can be helpful to scale-up performance level of working hospital managers. Recruiting healthcare management graduates who have primary needed skills and competencies to administrate the hospitals can play an important role in improvement of performance at the organizational and individual level. At the academic level, according to the proposed model and the importance of its dimensions, curriculum of healthcare management can be revised. Further research is needed to investigate the gap between the skills and performance of hospital managers and association between them. Also future study can use multi-source assessment method such as 360 degree feedback model to evaluate performance of hospital managers based on different assessors’ views to ensure more reality and validity of evaluation process.

## 5. Conclusion

Hospital managers play an important role in organizational success. The findings of this study demonstrate that there is a great need for the further improvement of hospital managers in regards to performance assessment. The use of appropriate dimensions and criteria for performance, prioritizing them and evaluating the performance of hospital managers using appropriate techniques, can play an effective role in the selection of qualified managers, identifying strengths and weaknesses in performance and continuous improvement of them. Our study suggests a multifaceted and holistic model using combination of applicable techniques such as FAHP and FTOPSIS, as an effective solution to assess hospital managers’ performance. The study also highlighted the strength and weaknesses of hospital managers’ performance in each of the dimensions and sub-dimensions compare with other colleagues that are helpful for them in professional progress and development. Finally the proposed model and instrument can be used within the hospitals or by outside assessors affiliated with the Ministry of Health and Medical Education (MOHME) as a standard PA instrument.

## References

[ref1] Akbari F, Tofighi S, Torabi A, Arab M (2005). Survey of relationship between leadership style and conflict management of hospital managers of Lorestan University of Medical Sciences. Yafteh Journal.

[ref2] AKKOÇ S, Vatansever K (2013). Fuzzy performance evaluation with AHP and TOPSIS methods: Evidence from Turkish banking sector after the global financial crisis. Eurasian Journal of Business and Economics.

[ref3] American College of Healthcare Executives (2015). ACHE healthcare executive competencies assessment tool.

[ref4] Anderson P, Pulich M (2002). Managerial competencies necessary in today’s dynamic health care environment. Health Care Management.

[ref5] Aozora Bank, Public Relations Department (2002). Hospital management assessment project.

[ref6] Avazpour R, Ebrahimi E, Fathi M. R (2013). A 360 Degree Feedback Model for Performance Appraisal Based on Fuzzy AHP and TOPSIS. International Journal of Economy, Management and Social Sciences.

[ref7] Calhoun J. G, Dollett L, Sinioris M. E, Wainio J. A, Butler P. W, Griffith J. R (2008). Development of an Interprofessional Competency Model for Healthcare Leadership. Journal of Healthcare Management.

[ref8] Chadwell I, Bhitrakoti R, Khadka R (2012). Measuring Management Training Needs of Hospital Managers in Nepal. Journal of Nepal Medical Association.

[ref9] Dadgar E, Janati A, Tabrizi J. S, Asghari-Jafarabadi M, Barati O (2012). Iranian Expert Opinion about Necessary Criteria for Hospitals Management Performance Assessments. Health Promotion Perspectives.

[ref10] Deng H (1999). Multi-criteria analysis with fuzzy pair-wise comparison. International Journal of Approximate Reasoning.

[ref11] Fang C. H, Chang S. T, Chen G. L (2010). Competency development among Taiwanese healthcare middle manager: A test of the AHP approach. African Journal of Business Management.

[ref12] Fazli S, Azar A (2002). Designing a Mathematical model of manager performance evaluation using Data Envelopment Analysis. Modarres Journal.

[ref13] Gharaei R (2004). Evaluating of managers competencies with 360 degree feedback method (Unpublished master’s thesis in Persian).

[ref14] Guo K. L (2002). Roles of managers in academic health centers: Strategies for the managed care environment. Health Care Manager.

[ref15] Islam R, Rasad S, b, M (2006). Employee performance evaluation by the AHP: A case study. Asia Pacific Management Review.

[ref16] Karsak E. E (2002). Distance-based fuzzy MCDM approach for evaluating flexible manufacturing system alternatives. International Journal of Production Research.

[ref17] Khadka D. K, Gurung M, Chaulagain N (2013). Managerial competencies: A survey of hospital managers working in Kathmandu valley, Nepal. Journal of Hospital Administration.

[ref18] Kuo M. S, Tzeng G. H, Huang W. C (2007). Group decision making based on concepts of ideal and anti-ideal points in fuzzy environment. Mathematical and Computer modeling.

[ref19] Landry A. Y, Stowe M, Haefner J (2012). Competency assessment and development among health-care leaders: Results of a cross-sectional survey. Health Services Management Research.

[ref20] Liang Z, Leggat S. G, Howard P. F, Koh L (2013). What makes a hospital manager competent at the middle and senior levels. Australian Health Review.

[ref21] Lockhart W, Backman A (2009). Health care management competencies: identifying the GAPs. Healthcare Management Forum.

[ref22] Mac Kinnon N. J, Chow C, Kennedy P, Persaud D, Metge C, Sketris I (2005). Management competencies for Canadian health executives: Views from the field. Healthcare Management Forum, Elsevier.

[ref23] Mohd-Shamsudin F, Chuttipattana N (2012). Determinants of managerial competencies for primary care managers in Southern Thailand. Journal of Health Organization and Management.

[ref24] Nikoukar S, Ketabi S, Moazam E (2011). A Combined Model of Data Envelopment Analysis (DEA) and Analytical Hierarchy Process (AHP) in ranking of hospital managers. Health Information Management.

[ref25] Patil S. K, Kant R (2014). A fuzzy AHP-TOPSIS framework for ranking the solutions of Knowledge Management adoption in Supply Chain to overcome its barriers. Expert Systems with Applications.

[ref26] Pillay R (2008). Managerial competencies of hospital managers in South Africa: A survey of managers in the public and private sectors. Human Resources for Health.

[ref27] Pillay R (2010). The skills gap in hospital management: A comparative analysis of hospital managers in the public and private sectors in South Africa. Health Services Management Research.

[ref28] Saaty T. L (1980). The analytic hierarchy process.

[ref29] Shahkordi H (2003). Survey and determine the performance evaluation criteria of power distribution companies (Unpublished master’s thesis in Persian).

[ref30] Shewchuk R, O’Connor S, Fine D (2005). Building an understanding of the competencies needed for health administration practice. Journal of Healthcare Management.

[ref31] Stefl M. E (2008). Common competencies for all healthcare managers: The Healthcare Leadership Alliance model. Journal of Healthcare Management.

[ref32] Sun C. C (2010). A performance evaluation model by integrating fuzzy AHP and fuzzy TOPSIS methods. Expert Systems with Applications.

[ref33] Torfi F, Farahani R. Z, Rezapour S (2010). Fuzzy AHP to determine the relative weights of evaluation criteria and Fuzzy TOPSIS to rank the alternatives. Applied Soft Computing.

[ref34] Tsai H. Y, Chang C. W, Lin H. L (2010). Fuzzy hierarchy sensitive with Delphi method to evaluate hospital organization performance. Expert Systems with Applications.

[ref35] Van Laarhoven P. J. M, Pedrcyz W (1983). A fuzzy extension of Saaty’s priority theory. Fuzzy Sets and Systems.

[ref36] Wongprasit N (2014). The Leadership Competencies Model of Private Hospital Directors in Thailand. HRD Journal.

[ref37] Yang T, Hung C. C (2007). Multiple-attribute decision making methods for plant layout design problem. Robotics and Computer-Integrated Manufacturing.

[ref38] Zadeh L. A (1965). Fuzzy sets. Information and Control.

